# Supplementary material for: A Generalized Method for the Synthesis of Carbon-Encapsulated Fe3O4 Composites and Its Application in Water Treatment
Source: Molecules. 2022 Oct 11;27(20):6812. doi: 10.3390/molecules27206812 (PMC9607371; doi:10.3390/molecules27206812)
Supplement: Supplementary file 1 [file molecules-27-06812-s001.zip › molecules-1940795-supplementary.pdf]

molecules

Supplementary Materials

A Generalized Method for the Synthesis of Carbon-Encapsulated Fe_3O_4 Composites and Its Application in Water Treatment

Shengxia Duan [1,2], Jian Liu [3*], Yanling Pang [1*], Feng Lin [1], Xiangyan Meng [1], Ke Tang [1] and Jiaxing Li [2]

1 Department of Chemistry and Engineering, Heze University, Heze 274500, China
2 CAS Key Laboratory of Photovoltaic and Energy Conservation Materials, Institute of Plasma Physics, Chinese Academy of Sciences, Hefei 230031, China
3 Department of Agriculture and Bioengineering, Heze University, Heze 274500, China
* Correspondence: liujianscholar@163.com (J.L.); yanling19972004@126.com (Y.P.)

Synthesis of C@Fe_3O_4 Nanodisks and C@Fe_3O_4 Nanorparticles

C@Fe_3O_4 nanodisks were fabricated through a three-step process. (1) α-Fe_2O_3 nanodisks were firstly obtained by a hydrothermal method. Specifically, 1 mmol of $FeCl_3{\cdot}6H_2O$ and 2 mmol of $Na_2B_4O_7{\cdot}10H_2O$ were added into 40 mL of water under vigorous stirring; then, 8 mmol of NaOH(s) were added into the above solution stirring for about 10 min; after that, the whole mixture was transferred into a Teflon-lined stainless steel autoclave for hydrothermal treatment at 140 °C for 12 h. As the autoclave cooled to room temperature naturally, the precipitates *H1* were separated by centrifugation, washed with distilled water and absolute ethanol, and dried in vacuum at 60 °C for 12 h. (2) The α-Fe_2O_3/carbon shell beads were prepared by an ionic polymerization route. In a typical procedure, 1.5 g of sodium alginate powder was dissolved in 100 mL deionized water at 353.15 K for 12 h, resulting in a transparent and viscous solution. A sample of 1.5 g of α-Fe_2O_3 nanodisks was then added to the above viscous solution and stirred for 30 min. The aqueous solution containing sodium alginate and α-Fe_2O_3 nanodisks was slowly added dropwise to 0.18 $mol{\cdot}kg^{-1}$ $CaCl_2$ solution using a syringe. The drops gelled into magnetic beads when they contacted the $CaCl_2$ solution. Finally, the resulting beads were washed several times with distilled water and dried in vacuum at 60 °C for 12 h. [JCED-2011-56-3475]. (3) The product obtained above was treated at 400 °C for 2 h in a tube furnace with a heating rate of 1 $°C{\cdot}min^{-1}$ under Ar atmosphere. The sample were dealt with acid treatment and washed several times with distilled water and dried in vacuum at 60 °C for 12 h. After that treatment, the C@Fe_3O_4 nanodisks were obtained by the above processes. The synthesis procedure of C@Fe_3O_4 nanoparticles is similar to that of C@Fe_3O_4 nanodisks, except that the precusrsor α-Fe_2O_3 nanodisks has changed into the commercially available α-Fe_2O_3 nanoparticles.

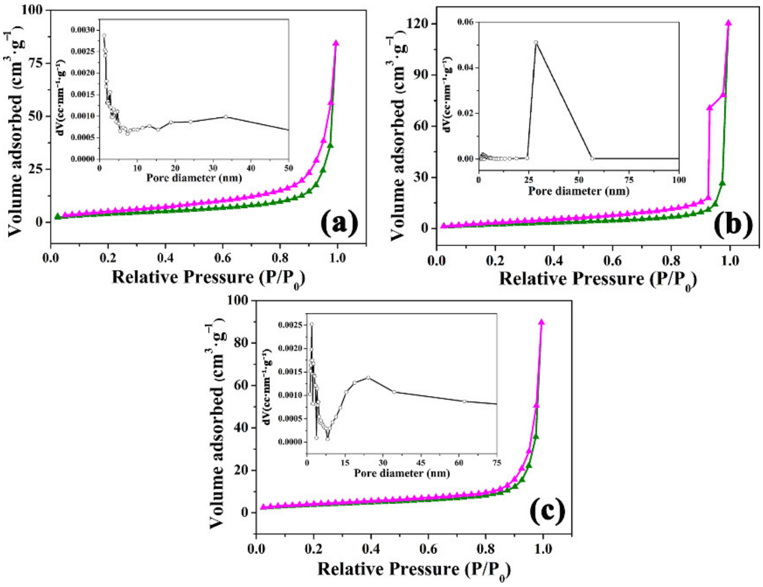

Figure S1. Nitrogen adsorption/desorption isotherm and Barrett–Joyner–Halenda (BJH) pore size distribution plot (inset) of (**a**) α-Fe_2O_3 nanorod, (**b**) α-Fe_2O_3 nanodisk and (**c**) α-Fe_2O_3 nanoparticle.

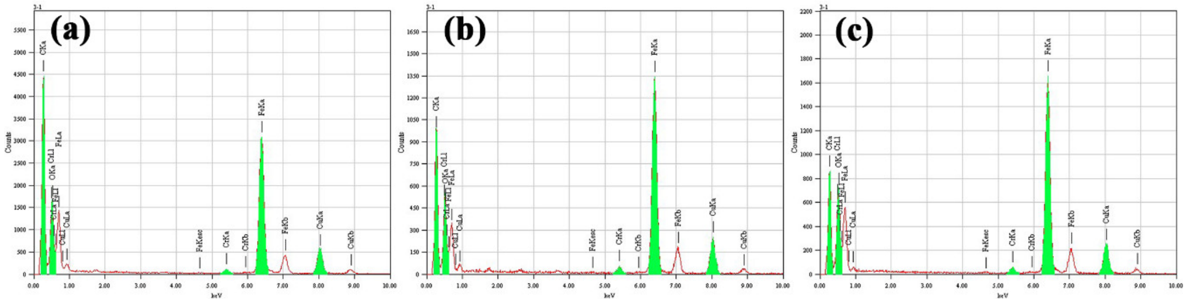

Figure S2. Corresponding EDS examination of the C@Fe_3O_4 composites (**a**) nanorod, (**b**) nanodisk and (**c**) nanoaparticles.

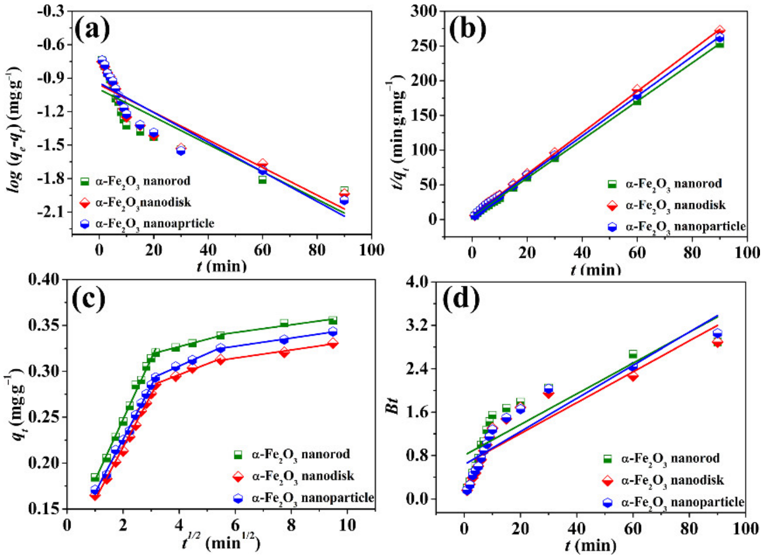

Figure S3. (**a**) Plot of log(q_e-q_t) vs. t for adsorption of MB onto α-Fe_2O_3 precursors by using the pseudo-first-order kinetic model; (**b**) Plot of t/q_t vs. t for adsorption of MB onto α-Fe_2O_3 precursors by using the pseudo-second-order kinetic model; (**c**) Plot of q_t vs. $t^{1/2}$ for adsorption of MB onto α-Fe_2O_3 precursors; (**d**) Plot of Bt vs. t for adsorption of MB onto α-Fe_2O_3 precursors.

Table S1. Parameters of intra-particle diffusion and Boyd kinetic models for the adsorption of MB onto C@Fe_3O_4 composites.

Samples	Models	Intra-Particle Diffusion (g·mg·min$^{-1/2}$)						Boyd Model	
C@Fe_3O_4 nanorods	Parameters	k_1	4.742	k_2	3.136	k_3	0.3673	R^2	0.9290
		R^2	0.9476	R^2	0.9632	R^2	0.7527		
C@Fe_3O_4 nanodisks	Parameters	k_1	2.394	k_2	0.9490	k_3	0.6778	R^2	0.9572
		R^2	0.9649	R^2	0.9995	R^2	0.9613		
C@Fe_3O_4 nanoparticles	Parameters	k_1	7.771	k_2	0.7173	k_3	0.3460	R^2	0.8464
		R^2	0.9888	R^2	0.9733	R^2	0.9938		

Table S2. Parameters of pseudo-first-order and pseudo-second-order for the adsorption of MB onto α-Fe_2O_3 samples.

Samples	Models	Pseudo-First-Order		Pseudo-Second-Order	
α-Fe_2O_3 nanorods	Parameters	k_1 (min^{-1})	0.0284	k_2 (g·mg·min^{-1})	0.8670
		q_e (mg·g^{-1})	0.1000	q_e (mg·g^{-1})	0.3613
		R^2	0.7452	R^2	0.9999
α-Fe_2O_3 nanodisks	Parameters	k_1 (min^{-1})	0.0285	k_2 (g·mg·min^{-1})	0.7373
		q_e (mg·g^{-1})	0.1102	q_e (mg·g^{-1})	0.3357
		R^2	0.8021	R^2	0.9997
α-Fe_2O_3 commercially available	Parameters	k_1 (min^{-1})	0.0306	k_2 (g·mg·min^{-1})	0.7020
		q_e (mg·g^{-1})	0.1142	q_e (mg·g^{-1})	0.3490
		R^2	0.8340	R^2	0.9997

Table S3. Parameters of intra-particle diffusion and Boyd kinetic models for the adsorption of MB ontoα-Fe_2O_3 samples.

Samples	Models	Intra-Particle Diffusion (g·mg·min$^{-1/2}$)						Boyd Model	
α-Fe_2O_3 nanorods	Parameters	k_1	0.0662	k_2	0.0081	k_3	0.0042	R^2	0.7452
		R^2	0.9934	R^2	0.9995	R^2	0.8562		
α-Fe_2O_3 nanodisks	Parameters	k_1	0.0572	k_2	0.0118	k_3	0.0044	R^2	0.8021
		R^2	0.9952	R^2	0.9817	R^2	0.9611		
α-Fe_2O_3 commercially available	Parameters	k_1	0.0582	k_2	0.0135	k_3	0.0044	R^2	0.8340
		R^2	0.9949	R^2	0.9925	R^2	0.9940		

Table S4. BET surface area and the adsorption capacity of the three C@Fe_3O_4 composites.

Adsorbent Sample	Pore Size (nm)	Surface Area (m^2·g^{-1})	Adsorption Capacity (mg·g^{-1})
C@Fe_3O_4 nanorod	3.9442	26.682	34.54
C@Fe_3O_4 nanodisk	3.9448	31.407	32.94
C@Fe_3O_4 nanoparticle	3.9446	27.003	33.64
